# Supplementary material for: Soil gas radon and soil permeability assessment: Mapping radon risk areas in Perak State, Malaysia
Source: PLoS One. 2021 Jul 28;16(7):e0254099. doi: 10.1371/journal.pone.0254099 (PMC8318270; doi:10.1371/journal.pone.0254099)
Supplement: S1 File — (DOCX) [file pone.0254099.s001.docx]

Minimal Data set

| Sampling Location Coordinates (0°) | | Radon in soil gas (kBq m^-3^) | Soil permeability (m^2^) |
| --- | --- | --- | --- |
| Longitude | Latitude |  |  |
| 101.4917 | 3.7111 | 1.95 | 1.2E-13 |
| 101.4814 | 3.7250 | 0.41 | 1.1E-13 |
| 101.4736 | 3.7236 | 0.105 | 1.8E-13 |
| 101.4000 | 3.8214 | 1.5 | 2.0E-13 |
| 101.3997 | 3.8311 | 1.009 | 2.2E-13 |
| 101.4094 | 3.8250 | 1.39 | 2.6E-13 |
| 101.2639 | 4.1933 | 1.2 | 4.7E-13 |
| 101.1608 | 4.2981 | 14 | 7.5E-13 |
| 101.1586 | 4.3064 | 12.9 | 4.5E-12 |
| 101.1558 | 4.3086 | 10.1 | 5.2E-14 |
| 101.1542 | 4.3092 | 75.75 | 1.8E-12 |
| 101.1347 | 4.4406 | 15.75 | 2.4E-12 |
| 101.1514 | 4.6300 | 1.2 | 1.0E-13 |
| 101.1383 | 4.6325 | 10.452 | 2.4E-13 |
| 101.1103 | 4.6133 | 1.009 | 5.2E-12 |
| 101.1097 | 4.6492 | 21.12 | 1.1E-12 |
| 100.9358 | 5.0206 | 115.98 | 2.6E-13 |
| 100.9358 | 5.0150 | 10.4 | 5.2E-14 |
| 100.9461 | 5.0064 | 10.21 | 5.2E-14 |
| 100.9628 | 4.8431 | 10.2 | 5.2E-14 |
| 100.9250 | 4.7719 | 1.02 | 5.2E-14 |
| 101.1256 | 5.4197 | 1.005 | 5.2E-14 |
| 101.1261 | 5.4269 | 1.05 | 5.2E-14 |
| 101.117 | 5.4261 | 2.93 | 1.1E-13 |
| 101.1064 | 5.4272 | 1.004 | 5.2E-14 |
| 101.0972 | 5.4406 | 46.67 | 1.7E-12 |
| 100.6878 | 5.2119 | 20.16 | 3.4E-12 |
| 100.6858 | 5.2100 | 11.8 | 5.2E-14 |
| 100.6858 | 5.2042 | 12.16 | 5.2E-14 |
| 100.4706 | 5.1061 | 13.01 | 5.2E-14 |
| 100.4783 | 5.1128 | 1.005 | 5.2E-14 |
| 100.4611 | 5.1022 | 1.21 | 5.2E-14 |
| 100.4883 | 5.1170 | 0.687 | 5.2E-14 |
| 100.4967 | 5.0825 | 1.002 | 5.2E-14 |
| 100.3928 | 5.0797 | 0.798 | 5.2E-14 |
| 100.3939 | 5.0792 | 1.002 | 5.2E-14 |
| 100.6189 | 4.9208 | 0.588 | 5.2E-14 |
| 100.4950 | 5.0861 | 0.369 | 5.2E-14 |
| 100.5994 | 4.9253 | 0.379 | 5.2E-14 |
| 100.7525 | 4.8528 | 3.05 | 5.2E-13 |
| 100.7453 | 4.8536 | 1.102 | 5.2E-14 |
| 100.7094 | 4.8414 | 1.003 | 5.2E-14 |
| 100.7503 | 4.8261 | 9.813 | 5.2E-14 |
| 100.7344 | 4.5589 | 434.5 | 1.5E-13 |
| 100.6758 | 4.5758 | 2.317 | 1.8E-13 |
| 100.6358 | 4.5042 | 1.068 | 5.2E-14 |
| 100.6733 | 4.2053 | 33.4 | 1.4E-13 |
| 100.6511 | 4.1886 | 1.118 | 5.2E-14 |
| 100.6461 | 4.1825 | 1.006 | 5.2E-14 |
| 100.9303 | 4.4711 | 1.003 | 5.2E-14 |
| 100.9197 | 4.4736 | 26.925 | 1.1E-12 |
| 100.9242 | 4.4725 | 2.91 | 5.2E-14 |
| 100.9853 | 4.3617 | 13.4 | 3.4E-12 |
| 100.9914 | 4.3586 | 26.65 | 1.4E-12 |
| 100.9828 | 4.3289 | 1.75 | 5.2E-14 |
| 100.9500 | 4.3250 | 30.7 | 2.9E-13 |
| 100.9508 | 4.3225 | 77.6 | 8.0E-13 |
| 100.9544 | 4.3283 | 1.51 | 5.2E-14 |
| 100.9936 | 4.3567 | 1.37 | 5.2E-14 |
| 100.9950 | 4.3542 | 1.145 | 5.2E-14 |
| 100.9267 | 4.1908 | 19.6 | 1.3E-13 |
| 100.9375 | 4.1842 | 37.6 | 2.2E-13 |
| 101.0317 | 4.0953 | 106.5 | 1.1E-12 |
| 101.0444 | 4.0719 | 2.14 | 5.2E-14 |
| 101.0433 | 4.0317 | 19.9 | 2.0E-12 |
| 100.7144 | 3.9029 | 2.018 | 1.7E-12 |
| 100.7175 | 3.9089 | 0.498 | 4.1E-13 |
| 100.7186 | 3.9036 | 27.6 | 1.1E-12 |
| 100.7761 | 3.9061 | 1.78 | 5.2E-14 |
| 100.7814 | 3.9022 | 1.791 | 5.2E-14 |
