# Supplementary material for: Soil gas radon and soil permeability assessment: Mapping radon risk areas in Perak State, Malaysia
Source: PLoS One. 2021 Jul 28;16(7):e0254099. doi: 10.1371/journal.pone.0254099 (PMC8318270; doi:10.1371/journal.pone.0254099)
Supplement: Attachment — Submitted filename: 10Jan2021_Response to Reviewers.docx [file pone.0254099.s002.docx]

Manuscript Number: PONE-D-20-31278

Response to reviewers’ comment

Academic editor

1. Please ensure that your manuscript meets PLOS ONE's style requirements, including those for file naming. The PLOS ONE style templates can be found at

https://journals.plos.org/plosone/s/file?id=wjVg/PLOSOne_formatting_sample_main_body.pdf and

https://journals.plos.org/plosone/s/file?id=ba62/PLOSOne_formatting_sample_title_authors_affiliations.pdf

*Authors reply:*

*Thanks for your suggestion, please note that we have revised the manuscript format accordingly.*

2. In your Methods section, please provide additional information regarding the permits you obtained to collect samples for the present study. Please ensure you have included the full name of the authority that approved the field site access and, if no permits were required, a brief statement explaining why.

*Authors reply:*

*Thanks for your suggestion, please note that there is no permit required for the on-ground radon survey as the sampling locations are located or selected in personal territory areas or highly risk areas. On top of that, this is government funding work and special privilege is granted for the researcher. A brief statement is given in methodology to address these comments.*

3. We note that Figures 1, 3, 7, and 8 in your submission contain map images which may be copyrighted. All PLOS content is published under the Creative Commons Attribution License (CC BY 4.0), which means that the manuscript, images, and Supporting Information files will be freely available online, and any third party is permitted to access, download, copy, distribute, and use these materials in any way, even commercially, with proper attribution. For these reasons, we cannot publish previously copyrighted maps or satellite images created using proprietary data, such as Google software (Google Maps, Street View, and Earth). For more information, see our copyright guidelines: http://journals.plos.org/plosone/s/licenses-and-copyright.

We require you to either (1) present written permission from the copyright holder to publish these figures specifically under the CC BY 4.0 license, or (2) remove the figures from your submission:

(1) You may seek permission from the original copyright holder of Figures 1, 3, 7, and 8 to publish the content specifically under the CC BY 4.0 license.

We recommend that you contact the original copyright holder with the Content Permission Form ([http://journals.plos.org/plosone/s/file?id=7c09/content-permission-form.pdf](about:blank)) and the following text:

“I request permission for the open-access journal PLOS ONE to publish XXX under the Creative Commons Attribution License (CCAL) CC BY 4.0 ([http://creativecommons.org/licenses/by/4.0/](about:blank)). Please be aware that this license allows unrestricted use and distribution, even commercially, by third parties. Please reply and provide explicit written permission to publish XXX under a CC BY license and complete the attached form.”

Please upload the completed Content Permission Form or other proof of granted permissions as an "Other" file with your submission.

In the figure caption of the copyrighted figure, please include the following text: “Reprinted from [ref] under a CC BY license, with permission from [name of publisher], original copyright [original copyright year].”

(2) If you are unable to obtain permission from the original copyright holder to publish these figures under the CC BY 4.0 license or if the copyright holder’s requirements are incompatible with the CC BY 4.0 license, please either i) remove the figure or ii) supply a replacement figure that complies with the CC BY 4.0 license. Please check copyright information on all replacement figures and update the figure caption with source information. If applicable, please specify in the figure caption text when a figure is similar but not identical to the original image and is therefore for illustrative purposes only.

*Authors reply:*

*Thanks for your suggestion, please note that all Fig. 1,3,7,8 are original work from authors, digitised images which produced from ArcGIS software. On top of that the geological map of Peninsular Malaysia is free for educational and research purpose. Please refer to our communication email with the officer at the Department of Mineral and Geoscience Malaysia under the Ministry of Energy and Natural Resources for approving our request to use the Geological Maps of Perak. The acknowledgement to this department has been added to express our gratitude on their cooperation.*

Reviewer #1

1. A large fraction of the observed data on soil gas radon concentration seem to be extraordinarily low (more in the range of outdoor air rather than soil gas). Please give a physical reasoning for these large amount of observations with very low values.

*Authors reply:*

*Thanks for your suggestion, please note that this could occur as a result of several factors such as soil permeability, particle size, porosity, water content near saturation level, soil comprising of clay, exhalation, advection, temperature and diffusion.*

*Alonso, H., Rubiano, J. G., Guerra, J. G., Arnedo, M. A., Tejera, A., & Martel, P. (2019). Assessment of radon risk areas in the Eastern Canary Islands using soil radon gas concentration and gas permeability of soils. . Science of the Total Environment, 664(2019), 449-460. doi:.* [*https://doi.org/10.1016/j.scitotenv.2019.01.411*](https://doi.org/10.1016/j.scitotenv.2019.01.411)

2. The interpolation method (empirical bayesian kriging) is not described at all. Please give a brief description. Also, please elaborate why you have chosen this interpolation technique (which is rather novel and sophisticated) and not a simpler one such as a more basic kriging alternatives. What are the advantages of EBK?

*Authors reply:*

*Thanks for your observation, please note that the description of EBK model, the explanation of why I chose this interpolation technique, has been added as a section. The advantage of EBK include: (a)It requires minimal interactive modelling (b) It gives more accurate standard errors of prediction than other kriging methods (c) It allows accurate predictions of moderately nonstationary data and (d) More accurate method for small data set than other Kriging methods.*

Krivoruchko, K. (2012). Empirical Bayesian Kriging Implemented in ArcGIS Geostatistical Analyst. *ArcUser Fall 2012 Software Development Team, Esri.*

3. How was cross-validation conducted? Please give details on fold assignment. Did you consider spatial autocorrelation in the observational data during cross-validation? What is the range of spatial auto-correlation in your data?

*Authors reply:*

*Thanks for your observation, please note that spatial autocorrelation was considered. The range of spatial auto-correlation in the data was inside the 95 percent interval.*

4. You showed a dependence of GRP on geology. Why do you not exploit this knowledge for spatial prediction/interpolation, e.g. by using geology as co-variable?

*Authors reply:*

*Thanks for your observation please note that, the EBK prediction/interpolation uses the geographical coordinates for the prediction/interpolation and it was done on a shape file of the geological map.*

5. The conclusion is only partly supported by your results. The high risk of granitic areas seems to be rather your hypothesis than your finding. Large parts of the high GRP areas are not in the granitic areas. For instance, a large fraction of the area covered with quaternary sediments (in the south and south-west of the study area) has a higher GRP than the granitic areas. This might be a consequence of the interpolation technique that you selected by not implementing geology as co-variable.

*Authors reply:*

*Thanks for your observation, please note that the conclusion has been rewritten to account for the outcropping of high GRP in quarternary and some low in granite areas.*

6. In many cases the language is not precise enough, partly ambiguous and grammatically not always sound.

*Authors reply:*

*Thanks for your observation, please note that the partly ambiguous and grammatical structures have been crossed checked and corrected.*

Minor comments
• L50: Rn is one of the leading causes for lung cancer, not for cancer in general

*Authors reply:*

*Thanks for your suggestion, please note that the sentence has been modified to reflect “*one of the leading causes of lung cancer”

• L54: “generate higher indoor Rn levels”

*Authors reply:*

*Thanks for your observation, please note that “generate higher indoor Rn levels” has been reframed to “the potential to generate higher indoor radon levels”*

• L 56: these methods are not geology-based. I guess you mean statistical methods (geostatistics, regression) using co-variable/proxy data

*Authors reply:*

*Thanks for your suggestion, please note that “geology based” has been corrected to “geostatistical-based”*

• L 57: Ariel? Please explain

*Authors reply:*

*Thanks for your observation, please note that “Ariel^”^ was a typographical error and has been corrected to “Aerial” and has to do with the design of semiconductor and scintillation gamma ray sensors integrated into aerial robotic plat forms, for the surveillance in hard to reach, hazardous areas while allowing for the measurements to be dynamically tracked and mapped*

• L76: What do you mean with “baseline” data? Unclear.

*Authors reply:*

*Thanks for your observation, please note that “baseline” has been deleted so that the sentence will be clear.*

• L 93: consider replacing geological “contexts” with “regions”

*Authors reply:*

*Thanks for your suggestion, please note that “contexts” has been replaced with “regions”*

• L96-98: The description of the sampling design is unclear to me. Please specify. Did you use the same fraction of measurements per geological region or did you sample preferentially in areas with expected higher values or higher geological heterogeneity?

*Authors reply:*

*Thanks for your observation, please note that the sampling has carried out such a manner that each geological formation will be included. It was difficult to get uniform number of sampling per geological formation because of the challenge of accessibility of some locations and denial of permission by some property owners.*

• Fig.1: What is the scale of the map? Please add sampling locations. Your geological classification in the legend is a mix of stratigraphic and lithologic classes. Please be consistent. Either stratigraphic or lithologic or lithostratigraphic.

*Authors reply:*

*Thanks for your suggestion, please note that the scale of the map has been inserted and sampling locations added to the map. The geological classification in the legend has been adjusted to lithologic.*

• Please indicate sampling locations also in Fig. 3, 7 & 8. This will help interpreting the maps.

*Authors reply:*

*Thanks for your suggestion, please note that the sampling locations have been indicated in Fig. 3, 7, & 8 as suggested.*

• Fig 2: please consider using smaller bins (e.g. 5 or 10 kBq/m³) for the histogram. In the box statistical indicators, a median of 0.15 seems to be unrealistic given my experience and also the data shown in the plot. Consider using standard deviation instead of error since you want to give a measure of dispersion in your data set rather than an error/uncertainty of an individual observation. There is no boxplot in this figure (figure caption)

*Authors reply:*

*Thanks for your suggestion, please note that smaller bins have been used for the histogram and standard deviation has been considered for measure of dispersion in the data. Box plot in figure caption has been deleted*

• I would recommend not using two decimal digits because it pretends an accuracy that is not existing in reality

*Authors reply:*

*Thanks for your suggestion, please note that the decimal digits have been adjusted.*

• L132: A soil gas Rn concentration of 0.01 kBq/m³ is a very low value. This is rather a Rn concentration observed in outdoor air than in soil (where Rn is continouosly produced). I would suspect such low values as erroneous measurements (e.g., due to contamination of your measured volume with outdoor air). If you consider these values to be realistic please elaborate on the processes in soil causing such low concentrations.

*Authors reply:*

*Thanks for your suggestion, please note that this could occur as a result of several factors such as soil permeability, particle size, porosity, water content near saturation level, soil comprising of clay, exhalation, advection, temperature and diffusion.*

*Alonso, H., Rubiano, J. G., Guerra, J. G., Arnedo, M. A., Tejera, A., & Martel, P. (2019). Assessment of radon risk areas in the Eastern Canary Islands using soil radon gas concentration and gas permeability of soils. . Science of the Total Environment, 664(2019), 449-460. doi:.* [*https://doi.org/10.1016/j.scitotenv.2019.01.411*](https://doi.org/10.1016/j.scitotenv.2019.01.411)

• L136: You performed log-transformation of your data prior to geostatistical modelling. How did you backtransform your data to the original scale after interpolation.

*Authors reply:*

*Thanks for your observation, please note that the backtransform is done using the geostatistical analyst tool in the ArcGIS software*

• L 166: “marine soils” are misleading for soils with marine sediments as parent material. Please consider revising.

*Authors reply:*

*Thanks for your suggestion, please note that the line has been revised.*

• Fig. 3. In this map a few apparent artefacts (e.g., in the east next to the label of Kelantan) are existent. Further, I suggest using even value breaks instead of giving two decimal digits.

*Authors reply:*

*Thanks for your suggestion, please note that the map has been adjusted and the two decimal digits values has been changed.*

• L 174ff: I suggest shifting the explanation of a boxplot into the caption of Fig4 because it breaks somewhat the flow in your text and it can be assumed that most readers are familiar with the concept of a boxplot.

*Authors reply:*

*Thanks for your suggestion, please note that the paragraph has been matched with the previous one and the general description of boxplot has been moved to the materials and method as a sub-section.*

• L180: The highest mean Rn conc. was found in Granite but the highest median in sed./metamorph. Please be more precise.

*Authors reply:*

*Thanks for your suggestion, please note that the confusing sentence has been revised by removing “mediume” & “highest value”.*

• Fig 4: Does Fig. show Rn data on the original scale or log-transformed data. If it shows data on the original scale, most values are exceptionally low which would require a physical explanation. How many samples do you have in each class? Please give n per class.

*Authors reply:*

*Thanks for your observation, please note that Rn data was on the original scale. The physical explanation is that the low values are because of low soil permeability, high humidity and other meteorological factors. The number of samples have been indicated.*

• L 211ff: the GRP is practically dimensionless since the log of k is used in eq. 1

*Authors reply:*

*Thanks for your observation, please note that the dimension has been corrected*

• L227: Please describe your geostatistical model. What are the parameters of your variogram?

*Authors reply:*

*Thanks for your suggestion, please note that the geostatistical model and the parameters of the variogram has been describe in the sub-section Empirical Bayesian Kriging (EBK).*

• L227: What is the reasoning for using 9 colour codes? If you do a classification in low, medium and high, it would be straightforward to use only 3 codes

*Authors reply:*

*Thanks for your suggestion, please note that the classification low, medium and high has been changed to 3 colour codes*

• L240: Please describe your cross-validation procedure. How did you assign observation to the testing fold? Random, stratified, spatially blocked?

*Authors reply:*

*Thanks for your suggestion, please note that the cross-validation procedure that was used has been described in the section as suggested.*

• Fig. 8: resolution of the figure is poor.

*Authors reply:*

*Thanks for your observation, please note that the resolution of the figure has been improved*.

• L259ff: your conclusion is not fully supported by your results. You claim that high GRP areas are mainly found in the granitic areas. In fact, the high GRP regions in Fig 7 and the outline of the granitic rocks (Fig1) do overlap only partly. I feel that this granitic regions being at higher risk is rather your hypothesis than your finding. Further, large parts of the high GRP areas are located in the quaternary sediments. This might be a consequence of their proximity to high GRP observations in the nearby granitic areas. If this is the case (I can only speculate on this issue because information on sampling locations is missing), I suggest considering the implementation of your knowledge (dependence of GRP on geology) in your spatial prediction/interpolation model (e.g., by using geology as co-variable).

*Authors reply:*

*Thanks for your suggestion, please note that the conclusion has been rewritten to account for the outcropping of high GRP in quarternary and some low in granite areas.*

• L 267: Why do you refer to a reference level of 300 Bq/m³? Your paper deals with the GRP, while the reference level of 300 Bq/m³ (or 100 Bq/m³or 200 Bq/m³) applies to indoor environments. The indoor reference level cannot be applied to your GRP estimates.

*Authors reply:*

*Thanks for your observation, please note that the line has been reconstructed in terms of the GRP only.*

Reviewer #2: 


Introduction
Lines 53-54: Please, check the definition of RPA, and clarify its relationship with indoor radon. “Radon Prone Area” is a term that describes an area where the probability of enhanced radon concentration is much higher than average. The establishment of such areas, by national authorities, can be based on geological conditions and/or the results of measuring campaigns of indoor radon concentration. See ICRP 1993 (Protection against radon-222 at home and at work. ICRP Publication 65. Paragraph 3.3)

*Authors reply:*

*Thanks for your observation, please note that the sentence with the term “Radon Prone Area” has been reframed to contain “the potential to generate higher indoor radon levels”*

Line 57: “Ariel” ?

*Authors reply:*

*Thanks for your observation, please note that “Ariel^”^ was a typographical error and has been corrected to “Aerial” and has to do with the design of semiconductor and scintillation gamma ray sensors integrated into aerial robotic plat forms, for the surveillance in hard to reach, hazardous areas while allowing for the measurements to be dynamically tracked and mapped*

Lines 67-68: High GRP indicates a high availability of geogenic radon that can enters indoor. Also, express clearly that GRP is related to Ra content (but also U content!) because it is a Rn source, whereas permeability (but also faults!) is related to Rn mobility.

*Authors reply:*

*Thanks for your suggestion, please note that the sentence has been reframed to include, “^238^U content” and “faults which are related to ^222^Rn mobility”.*

Line 69: I suggest to add here a short review of the methods previously used to map GRP.

*Authors reply:*

*Thanks for your suggestion, please note that a short review of the methods previously used to map GRP has been added.*

Lines 75-76: Please, summarize the results of previous studies.

*Authors reply:*

*Thanks for your suggestion, please note that summary of the results of previous studies are per the references numbers 26 – 33.*

Line 78: What data? Please, add description and reference.

*Authors reply:*

*Thanks for your observation, please note that it is referring to available data from literature on* *radon concentration studies in soil gas in Malaysia as per reference number 34.*

Line 82: A strict definition of “radon risk” should be presented.

*Authors reply:*

*Thanks for your suggestion, please note that “Radon risk” is herein defined as the probability or likelihood of harm or the severity of the harm from exposure to radon. The harm is mainly lung cancer, which is a painful and fatal disease*

*WHO. WHO Handbook on Indoor Radon: A Public health perspective. Geneva, Switzerland: WHO Press; 2009.*

2.0 Materials and Method
In this section, after the description of the study area and the analytical techniques, I expected to read a paragraph about the geostatistical technique used to map the GRP. Practically, the discussion is based on the maps elaborated using Empirical Bayesian Kriging (EBK), but the method description is absent. Please, add the description of EBK model and explain why you chose this interpolation method. What semivariogram did you select? What the maximum number of points in each local model? What software did you use? What is the resolution of your maps?

*Authors reply:*

*Thanks for your observation, please note that* *the description of EBK model, the explanation of why I chose this interpolation method, the semivariogram I selected, the maximum number of points in each local model, the software I used and the resolution of my maps has been added as a sub-section “Empirical Bayesian Kriging”.*

2.1 Study area and sampling points
Lines 92-95: The geological description is too scarce, please add more information. Faults and structural discontinuities may be important pathways for radon movement in the subsoil, so you should describe the tectonic frame of the study area.

*Authors reply:*

*Thanks for your suggestion, please note that more information has been added to the geological description. The tectonic frame of the study area has also been described as suggested.*

Line 96-97: please, add the location of sampling points in Fig.1. Also, calculate the sampling density. How many samples for each geological formation?

*Authors reply:*

*Thanks for your suggestion, please note that the sampling points have been added to figure 1. The samples in each geological formation were Quaternary (22), Sedimentary/Metamorphic rocks (26), Silurian (10), and Granite rocks (12) respectively.*

2.2 Measurement of radon in soil gas
Please, specify in what period of the year and meteorological conditions the measurements were performed.

*Authors reply:*

*Thanks for your observation, please note that measurements were carried out at low or no rainfall periods and moderate temperatures.*

Line 109: Did you use a drying tube to maintain the relative humidity at low values?

*Authors reply:*

*Thanks for your question, please note that the drying unit was used to maintain the relative humidity of the RAD7 at low values.*

Results and discussion

3.1 Activity concentration of radon in soil gas
Lines 131-132: for non-normal distribution, better to use geometric mean

*Authors reply:*

*Thanks for your suggestion, please note that the distribution standard deviation has been used instead in order to account for measure of dispersion.*

Lines 132-134: this description is does not fit to the histogram of fig. 2, probably a histogram with more classes will be better describe your data. Please, consider using a normal probability plot; it can help to recognize both anomalous samples and multiple populations in log-normally distributed data

*Authors reply:*

*Thanks for your suggestion, please note that a histogram with more bins have been used.*

Lines 152-153: here the Authors introduce for the first time the Empirical Bayesian Kriging; see the comment above.

*Authors reply:*

*Thanks for your observation, please note that Empirical Bayesian Kriging has now been introduced as a sub-section in the manuscript as earlier suggested.*

Lines 153-155: this description is unnecessary; better to specify how you have determined the class breaks (e.g., manual interval, quantile, natural breaks etc.)

*Authors reply:*

*Thanks for your observation, please note that lines 153 – 155 have been re-written in terms of how the class breaks were determined.*

Line 157: “ Manjung District”, and other localities cited later in the text (e.g., line 214), are unknown to non-Malaysian. Please, add it in the Figures.

*Authors reply:*

*Thanks for your suggestion, please note that “Manjung District” has replaced with* *the most western granitic region of the state”. It is not necessary to point out specific place. The rest of cited localities is removes and replaced with geological/cardinal direction.*

Lines 158-159: An explanation is needed of how this list of soil types is used and how these soils were identified. Did you identified these soils in field? Did you extrapolated from a pedological map? Is there a correlation between soil types and Rn concentration and/or permeability?

*Authors reply:*

*Thanks for your suggestion, please note that an explanation of how the list of soil types used and how these soils were identified has been added in the materials and method section.*

Lines 159-162: This statement causes some doubts. The Authors identified the area with the highest values of soil gas radon in Manjung District, which is located in the southwestern part of the state of Perak; here, granite rocks outcrops and therefore the Authors explain the high Rn values because it derived from such bedrock. However, the geological map of fig. 2 shows that granite rocks extensively outcrop in the eastern part of the study area, where the soil gas Rn concentrations are not particularly high (Fig.3). So, how the Authors explain these spatial distribution? Did you consider the presence of permeable structures such as fractures and/or faults?

*Authors reply:*

*Thanks for your observation, please note that this could occur as a result of several factors such as soil permeability, particle size, porosity, water content near saturation level, soil comprising of clay, exhalation, advection, temperature and diffusion. Moreover, most of the eastern part extensively outcropped by granite rocks were not accessible as the area is covered by thick forest and so were not sampled as seen in fig. 2 now that the sampling points have been indicated.*

Line 163: What mines/quarries are in the study area? Where they are located?

*Authors reply:*

*Thanks for your suggestion, please note that these were not considered in the scope of the study.*

Line 169: I suggest to add a table (or a box-plot) with the statistics (average values, ranges of values) of soil gas Rn concentration and permeability in different soil types, I think that it can be help the reader.

*Authors reply:*

*Thanks for your suggestion, please note that it is impossible to perform boxplot as the data per soil types (~ less than 7 samples per soil types) is too low, compared to geological influence which only 4 types.*

3.2 Radon activity concentration for each geological type
Sincerely, there is no reason to separate this paragraph from the previous one.
Lines 174-178: the general description of box-plot should be moved in the method section. A sub-paragraph describing statistical and geostatistical elaborations might be appropriate.

*Authors reply:*

*Thanks for your suggestion, please note that the paragraph has been matched with the previous one and the general description of boxplot has been moved to the materials and method section.*

3.3 Soil gas permeability
Lines 197-198: Please, add a map of spatial distribution of permeability, it could be very useful here. Please, also discuss how permeability influences radon in soil, in general and in your case study.
Table 1: Add the number of your samples that fall within each class of permeability.

*Authors reply:*

*Thanks for your suggestion, please note that a map of spatial distribution of permeability has been added, and how permeability influences radon in soil has been discussed, and the number of samples that fall within each class of permeability has been added to Table 1.*

3.4 Geogenic Radon Potential (GRP) and Radon prone areas
Line 210: “Many years of extensive research in the Czech Republic”…please, add references.

*Authors reply:*

*Thanks for your suggestion, please note that references have been added.*

Line 232: the title of the paragraph is “Geogenic Radon Potential (GRP) and Radon prone areas”, but where in the text the RPA are described?

*Authors reply:*

*Thanks for your observation, please note that, “Radon prone areas” has been described already in L53 – 54 in the introduction.*

Please, compare the map of GRP with the map of Terrestrial Gamma Dose Rate (TGDR) published by Ramli et al (2016) - Predicting terrestrial gamma dose rate based on geological and soil information: case study of Perak state, Malaysia. J. Radiol. Prot. 36(1), 20-36.

*Authors reply:*

*Thanks for your suggestion, please note that it is impossible to compare two different measurement units between map. TGDR map produced by Ramli et al 2016 is an in-situ data of absorbed dose in air (nGy/h) meanwhile GRP map is a statistical score map. On top of that, 70 preliminary sampling data of the present study is incompatible to the comprehensive TGRD data survey by Ramli et al.*

4.0 Conclusion
Lines 268-270: this concept should be emphasized in Discussion.

*Authors reply:*

*Thanks for your suggestion, please note that concept in Lines 268-270 has been emphasized in the Discussion section.*

Figures
Figure 1: add the location of sampling points, the prevailing tectonic lines, names of the district cited in the text

*Authors reply:*

*Thanks for your suggestion, please note that the sampling points have been added*

Figure 2: delete “and box plot” in the figure caption

*Authors reply:*

*Thanks for your suggestion, please note that “and box plot” has been deleted*

Figures 3 and 7: add the corresponding semivariograms.

*Authors reply:*

*Thanks for your suggestion, please note that corresponding semivariogram has been added*

Figure 4: what represent the blue dashed lines? How many samples for each geological type?

*Authors reply:*

*Thanks for your observation, please note that the blue lines are value range of 222Rn activity ranges 0 – 1, 1 – 10, 10 – 100 and 100 and above.*

Figure 5: add box plots classified according to geological formations and/or soil types.

*Authors reply*

*Thanks for your suggestion, please note that a box blot of soil gas permeability according to geological types have been added as figure 6.*

Figure 7: add a layer of urbanized areas, in order to evaluate if the areas of high RI are already urbanized or not yet; in the first case, the map could indicate the areas where it could be necessary monitoring the indoor radon concentration, in the second case the map could be a tool for future urbanistic plans.

*Authors reply:*

*Thanks for your suggestion, please note that for urbanised areas the indoor radon measurements are recommended.*
