# Supplementary material for: Soil gas radon and soil permeability assessment: Mapping radon risk areas in Perak State, Malaysia
Source: PLoS One. 2021 Jul 28;16(7):e0254099. doi: 10.1371/journal.pone.0254099 (PMC8318270; doi:10.1371/journal.pone.0254099)
Supplement: Attachment — Submitted filename: Response to Reviewers.docx [file pone.0254099.s003.docx]

PONE-D-20-31278R1
Soil gas radon and soil permeability assessment: Mapping radon risk areas in Perak State, Malaysia.
PLOS ONE

ACADEMIC EDITOR comments:
While the authors have addressed minor issues with the original submission, there are still problems with this paper. Mapping Rn is the main objective of the paper so the mapping method must be explained and justified but Reviewer #1 points to lingering issues with this. The problem with the geostatistical method may need the authors to enlist help from a specialist in this field.

*Authors reply:*

*Thanks for your observation/suggestion, please note that the geostatistical method of mapping Radon has been changed to Cokriging based on ordinary kriging. An appropriate semivariogram general equation of the model has been included.*

Review Comments to the Author

 Reviewer #1: Thanks to the authors for addressing all comments. Many of the minor issues related to grammar, style and figures have been mostly solved. However, the two fundamental issues of 1) Rn soil value range and the 2) the EBK predictive map are still unsolved from my point of view.
1. Soil Radon value range: I am still not convinced on the accuracy such low values (depth) which seem for most samples 1-2 orders of magnitude too low. You give median values (Fig. 4) between 0.1 and 1.0 kBq/m³ for the four different geological units. I have great doubts on the correctness of these data and rather suspect issues with your sampling procedure. I consider values below 10 kBq/m³ for soils with granitic parent material as rare and below 1 kBq/m³ as highly unlikely. In your study most of the values are below 1 kBq/m³. Based on my experience and the values reported in the literature these soils with granitic parent material are often reported to lie in the range 10 – 1000 kBq/m³. You correctly mention the various factors influencing soil Rn concentration such as soil physical properties, soil moisture, permeability etc. But, I cannot imagine that the interplay of these factors result in such a large number of very low values. Further, in contrast to your statement that low values may be associated with low permeability, I would rather expect the reverse pattern: low permeability decreases Rn transport (diffusion, advection) and, consequently, decreases Rn exhalation. This means at the same time increase of Rn accumulation in soils which is associated with higher Rn concentration.
I suggest participation in soil gas intercomparison measurements for quality assurance and to be able to exclude issues with your sampling procedure.

*Authors reply:*

*Thanks for your observation, please note that, the low Rn in soil values occurred as a result of an error in computing the average values from the raw data.*

*According to the RAD7 user manual, when radon is introduced into the chamber, at first there is no count. After a few minutes the counts begin in window A, which is polonium-218, a result of the decay of Rn-222. For the first five minutes or so the count rate increases, then begins to approach a steady level. After about 10 minutes, the polonium -218 daughter reaches close to equilibrium with the parent radon-222. The first two 5-minutes cycles should be ignored, while the next one or two cycles should be averaged to arrive at the radon concentration of the soil gas (Durridge RAD7 User manual, 2015): pp39 &67*

*In the earlier manuscript, the average was taken over six readings instead of four readings, as the first two readings are supposed to be excluded according to the RAD7 user manual. Find below a sample of the raw data printout in which the first two readings are 69.8 and 140 Bq m^-3^. respectively. An average of the six readings is 8710 Bq m^-3^ while excluding the first two readings and taking average of the four gives 13,013 Bq m^-3^ resulting to an underestimation of over 4,000Bq m^-3^. Thus, all the results have been recalculated accordingly.*

2. Your reasoning for selection of your mapping method EBK does not convince me. It seems that the selection of EBK was motivated by the ease of its implementation in the sense of an automatic data processing and map production. While this may be convenient, it is dangerous because the method seems to be rather treated as a black box.
You correctly emphasize the high importance of geology for spatial variability of Rn in soil. Giving this knowledge and the availability of the required data (geological map of your study region), why do you not implement this knowledge in your mapping procedure? At the current state, your interpolation completely ignores geological boundaries – you simply interpolate across those borders. Such a procedure cannot be assumed to provide accurate estimates since measurements in one geological unit cannot be used to produce predictions in another geological unit without consideration of geology as co-variable. From a geostatistical point of view, the range of spatial- autocorrelation varies throughout your study area. This issue manifests in the fact that your top hotspot region mainly results from a single very high value. This single high value spreads from the granitic area over a very large region into other geological units and just stops at the nearest observations. This is not plausible at all to me. I highly recommend implementing geology as co-variable for mapping soil Rn. This is feasible with EBK, but also with simpler geostatistical approaches (such co-kriging based on ordinary kriging).
Further, artefacts are still visible in the map in the north-east where no observations were conducted (I suggest to clip these areas because predictions are not supported by observations. This issue can also be seen in Fig. 11 (dark red region in the north-east). Moreover, the resolution (i.e., grid cell size, e.g. 1km * 1km) of the map is still unclear.

*Authors reply:*

*Thanks for your observations/suggestion, please note that the geostatistical method has been changed to Cokriging based on ordinary kriging, and that geology has been implemented as co-variable for the mapping soil gas radon. The grid cell size of the map is* $5km \times5km$

Reviewer #2: Dear Editor
The authors tried to respond the questions arose from the first review. However, some questions are still unresolved, therefore I propose a minor revision for further improving the manuscript. In detail:

L 86-87: Please, add a short description of the results of the cited references (references numbers 26 – 33) in the text of your manuscript.
 *Authors reply:*

*Thanks for your suggestion, please note that a short description of the results of references 26 -33 have been added in the revised manuscript.*

L 169: Please, I would like to know the resolution of the data, i.e., the dimension of each pixel/cell of your raster (e.g., 10 x 10 km or 30" x 30")
 *Authors reply:*

*Thanks for your suggestion, please note that we have added the dimensions of the pixel in the revised manuscript.* $5km \times5km$
L 203-205: you did not describe how the class breaks were determined. So, what classification method did you use (manual interval, equal interval, quantile, natural breaks, geometrical interval, standard deviation)?
 *Authors reply:*

*Thanks for your observation, please note that the class breaks were determined using the geometrical intervals and has been added to the revised manuscript.*

L 302 and Fig. 9: You wrote “A three-color code was used in categorizing the RI.” So I imagine that you have used the classification described between the lines 286-290 (i.e., low RI (GRP < 10), medium RI (10

*Authors reply:*

*Thanks for your observation, please note that Figure 9 has been re-labelled to reflect low RI (GRP < 10), medium RI (10 >GRP) and high RI (35<GRP)*

Fig.1 Legend of geology: the lithology is still confused with the age of geological formations. The text (L 107-113) has the same problem.
 *Authors reply:*

*Thanks for your observation, please note that, addition details of the geological formations have been added in lines 107-113.*

Fig 2. I suggest to increase the number of bins (not only increase the number of classes in the X axis)
 *Authors reply:*

*Thanks for your suggestion, please note that we have increased the number of bins in the Figure as suggested.*

Fig 9: add a layer of urbanized areas or the location of the main cities. I agree that indoor radon measurements are recommended for the urbanized areas, I simply would know if there are important cities in the regions characterized by medium to high RI.

*Authors reply:*

*Thanks for your suggestion, please note that the location of main cities have been added to the figure.*
